# Mining the biomass deconstructing capabilities of rice yellow stem borer symbionts

**DOI:** 10.1186/s13068-019-1603-8

**Published:** 2019-11-08

**Authors:** Rahul Singh, Joseph P. Bennett, Mayank Gupta, Medha Sharma, Danish Eqbal, Anna M. Alessi, Adam A. Dowle, Simon J. McQueen-Mason, Neil C. Bruce, Syed Shams Yazdani

**Affiliations:** 1 Microbial Engineering Group, International Centre for Genetic Engineering and Biotechnology, New Delhi, India; 2 DBT-ICGEB Centre for Advanced Bioenergy Research, International Centre for Genetic Engineering and Biotechnology, New Delhi, India; 3 Department of Biology, Centre for Novel Agricultural Products, University of York, York, UK; 4 Department of Biology, Bioscience Technology Facility, University of York, York, UK

**Keywords:** Rice yellow stem borer, Gut consortium, Microbial diversity, Targeted enrichment, Metaexoproteome, Carbohydrate-active enzymes, Xylanase, GH10 family

## Abstract

**Background:**

Efficient deconstruction of lignocellulosic biomass into simple sugars in an economically viable manner is a prerequisite for its global acceptance as a feedstock in bioethanol production. This is achieved in nature by suites of enzymes with the capability of efficiently depolymerizing all the components of lignocellulose. Here, we provide detailed insight into the repertoire of enzymes produced by microorganisms enriched from the gut of the crop pathogen rice yellow stem borer (*Scirpophaga incertulas*).

**Results:**

A microbial community was enriched from the gut of the rice yellow stem borer for enhanced rice straw degradation by sub-culturing every 10 days, for 1 year, in minimal medium with rice straw as the main carbon source. The enriched culture demonstrated high cellulolytic and xylanolytic activity in the culture supernatant. Metatranscriptomic and metaexoproteomic analysis revealed a large array of enzymes potentially involved in rice straw deconstruction. The consortium was found to encode genes ascribed to all five classes of carbohydrate-active enzymes (GHs, GTs, CEs, PLs, and AAs), including carbohydrate-binding modules (CBMs), categorized in the carbohydrate-active enzymes (CAZy) database. The GHs were the most abundant class of CAZymes. Predicted enzymes from these CAZy classes have the potential to digest each cell-wall components of rice straw, i.e., cellulose, hemicellulose, pectin, callose, and lignin. Several identified CAZy proteins appeared novel, having an unknown or hypothetical catalytic counterpart with a known class of CBM. To validate the findings, one of the identified enzymes that belong to the GH10 family was functionally characterized. The enzyme expressed in *E. coli* efficiently hydrolyzed beechwood xylan, and pretreated and untreated rice straw.

**Conclusions:**

This is the first report describing the enrichment of lignocellulose degrading bacteria from the gut of the rice yellow stem borer to deconstruct rice straw, identifying a plethora of enzymes secreted by the microbial community when growing on rice straw as a carbon source. These enzymes could be important candidates for biorefineries to overcome the current bottlenecks in biomass processing.

## Background

The use of lignocellulosic ethanol as a sustainable alternative to fossil fuel-derived transportation fuel or first generation biofuels depends upon consistent biomass availability and the economic viability of the bioethanol production process. Among all the lignocellulosic biomass available as potential feedstocks in lignocellulosic ethanol production, the availability of agricultural residues is attractive, as the amount produced on an annual basis is likely to increase in the future due to increased demand of crop production to fulfil the nutritional requirement of the rapidly growing world population. Rice straw, wheat straw, sugarcane bagasse, and corn stover are currently the most available agricultural residues, with rice straw being the most abundant (731 million tons) [1], totalling more than the sum of the other three crops (663 million tons) [2]. Rice straw also contains the least amount of lignin (one of the limiting factors towards making lignocellulosic ethanol cost competitive) when compared to all other abundantly available agricultural residues [3–5] making it a desirable choice as feedstock for lignocellulosic ethanol production [6–9]. Moreover, due to its limited suitability for other purposes due to its high silica content [10, 11], farmers usually burn the rice straw in the field wasting a potentially valuable resource, releasing emissions of black carbon, CO_2,_ and generating tropospheric ozone [12–14]. A major barrier in delivering cost effective lignocellulosic bioethanol is the availability of enzymes that can efficiently deconstruct each component of the plant cell wall. Indeed, none of the current formulations of biomass degrading enzymes fully meet the requirements of the biofuels’ industry [15]. To overcome these limitations, a diverse range of lignocellulosic degrading organisms are being explored for new enzyme activities, including insects, which have evolved to digest wider range of lignocellulosic substrates [16–18].

The type of enzymes required for effective deconstruction of biomass depends on the nature or structural component of their cell wall. There is no universal cocktail of enzymes that can effectively deconstruct each type of biomass and it is usually customized on the basis of biomass composition [19, 20]. Most enzymes used in commercial lignocellulosic ethanol production have been discovered from pure fungal or bacterial isolates [21]. In this paper, we describe the selective enrichment of a microbial consortium from the gut of a rice yellow stem borer (*Scirpophaga incertulas*) using rice straw as the sole carbon source. The yellow stem borer (YSB) is monophagous, i.e., it derives nutrition solely from stems of rice plants. It is, therefore, highly specialized to deconstruct the cell walls of rice plants into simple sugars [22]. Microbial communities residing in the gut of biomass degrading insects are known to interplay synergistically for comprehensive biomass deconstruction [23–26]. A metatranscriptomic and metaexoproteomic study was performed on a rice straw-enriched microbial community from rice stem borer larvae to investigate the CAZy proteins mediating the deconstruction of rice plant cell walls. Several new enzymes categorized to different CAZy classes were identified, one of which belonging to family GH10 was heterologously expressed in *E. coli* and its deconstruction ability towards the hemicellulose component of rice straw established.

## Results

### Microbial diversity of a rice yellow stem borer gut consortium

**Fig. 1:**
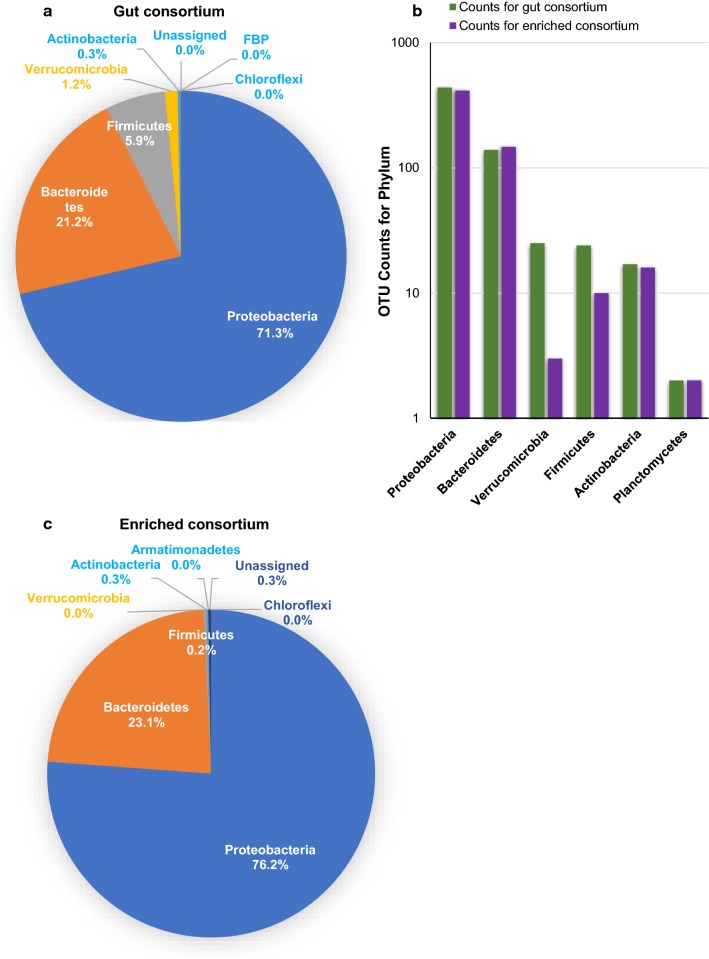
Rice yellow stem borer gut microbial community structure at the level of Phylum. Relative abundance of phylum in the **a** gut consortium and in the **c** enriched consortium. **b** Total number of Operational Taxonomy Unit (OTU) in the gut consortium and in the enriched consortium

**Fig. 2:**
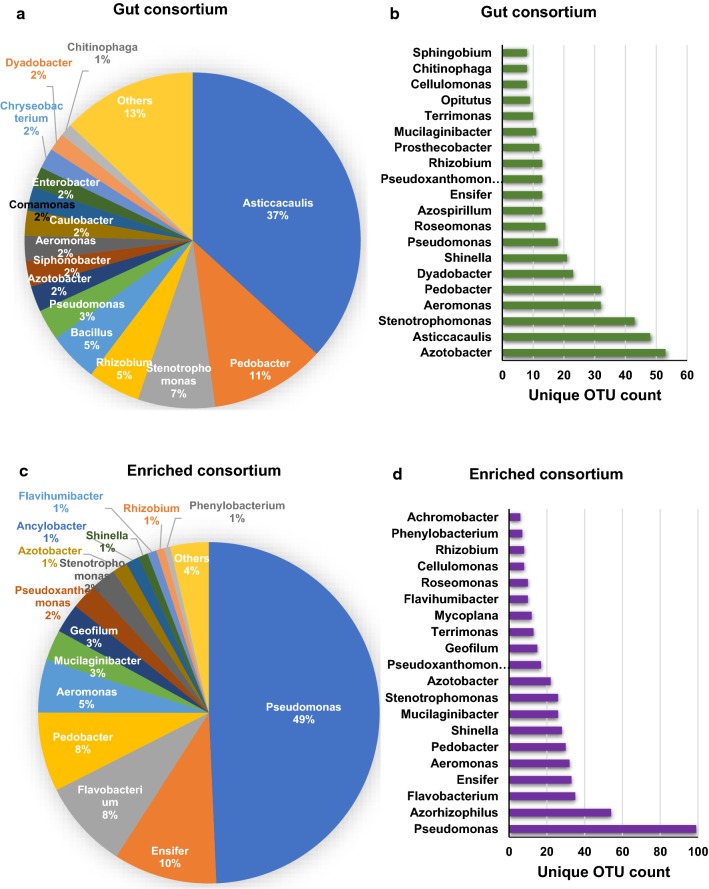
Rice yellow stem borer gut microbial community structure at the level of genus. Relative abundance of genus in the **a** gut consortium and in the **c** enriched consortium. Top 20 genera in terms of their unique OTUs detected in the **b** gut consortium and in the **d** enriched consortium

Rice yellow stem borer (YSB) larvae were collected from paddy fields and the larvae gut dissected to facilitate the collection of the gut fluid. 16S rRNA analysis of the microbial community present in the gut identified various operational taxonomic units (OTUs) that were affiliated to 178 genera belonging to 13 different phyla (Table 1). Proteobacteria, Bacteroidetes, Fermicutes, Verrucomicrobia, and Actinobacteria constituted greater than 99.5% of all phyla present in terms of relative abundance (Fig. 1a). A similar trend existed in terms of total number of unique OTUs detected under each category (Fig. 1b). The top 5 genera in terms of 16S rRNA gene abundance were *Asticcacaulis*, *Pedobacter*, *Stenotrophomonas*, *Rhizobium,* and *Bacillus*, which constituted 65% of all genera present in the gut (Fig. 2a). However, regarding higher diversities in the species detected within the genera, the top 5 genera detected were *Azotobacter*, *Asticcacaulis*, *Stenotrophomonas*, *Aeromonas,* and *Pedobacter* (Fig. 2b).

**Table 1:** Bacterial diversity in rice YSB gut consortium

S. No.	Name of phylum	Number of genera
1	Proteobacteria	101
2	Bacteroidetes	29
3	Actinobacteria	17
4	Firmicutes	14
5	Verrucomicrobia	6
6	Planctomycetes	3
7	Euryarchaeota	2
8	Acetobacteria	1
9	Chloroflexi	1
10	Armatimonadetes	1
11	Woesearchaeota	1
12	Crenarchaeota	1
13	Aquificae	1
Total genera		178

### Enrichment of a rice yellow stem borer gut microbial consortium

**Fig. 3:**
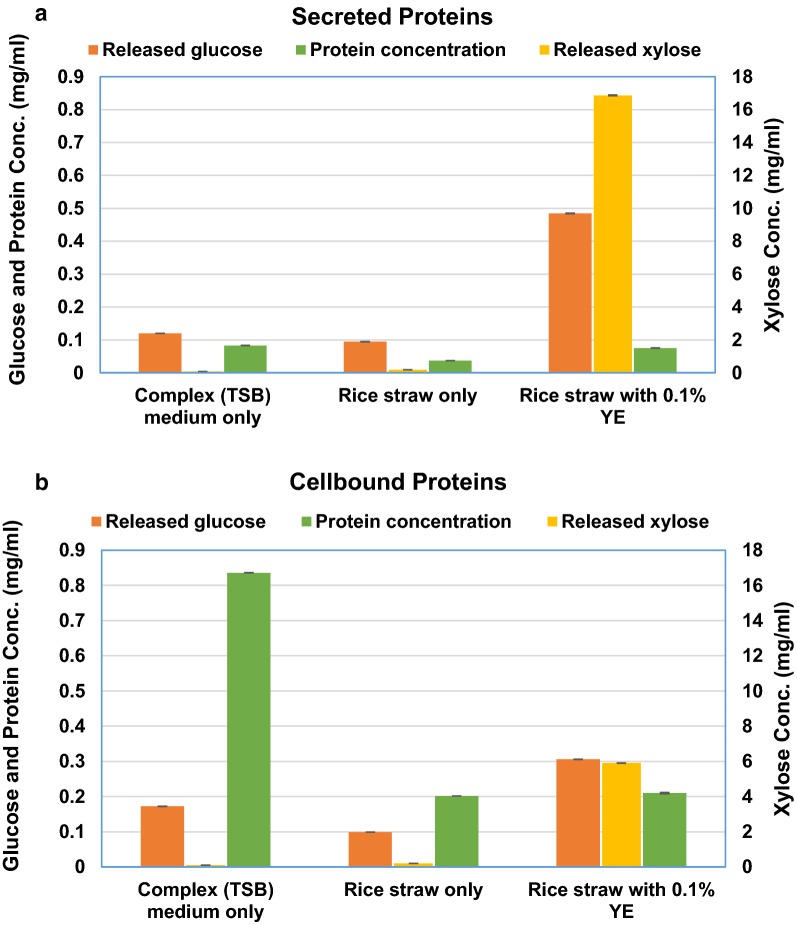
Evaluation of different culture conditions for biomass degrading enzyme production. Cultures were grown under various conditions, and secretory proteins (**a**) and cell bound protein extract (**b**) were evaluated for release of glucose and xylose using CMC and xylan as substrates, respectively. Data in **a** and **b** represent mean ± SD. *TSB* Tryptic Soya Broth, *YE* yeast extract

To enrich the isolated microbial consortium for rice straw degradation, serial sub-culturing was carried out in semi-defined medium containing chopped rice straw as the sole carbon source. Preliminary experiments were first performed to develop an optimized culture medium for the enrichment studies that was more suitable towards CAZy protein production. Three different media, i.e., (1) TSB, (2) rice straw in water plus salt, and (3) rice straw in water plus salt and 0.1% yeast extract, were investigated as described in “Methods”. TSB is a complex general-purpose medium that supports the growth of a wide variety of microorganisms (both gram positive as well as gram negative) was used for propagation of the maximum possible number of microorganisms in the culture for the production of the maximum possible types of lignocellulolytic enzymes. The other two media were selected for the maximum production of lignocellulolytic enzymes directed towards rice straw by providing rice straw as inducer. In Media-(3), small amount of yeast extract was also added to take care of any requirement of micro-nutrients for growth. Ghio et al. [27] also reported achievement of maximal cellulolytic and xylanolytic activity in a crude supernatant extract when bacteria were grown in minimal media with lignocellulosic substrate and yeast extract as nitrogen source. Moreover, successive passaging/sub-culturing of the consortium in the respective medium for the enrichment of lignocellulolyic enzymes is a common method and has been used in several studies [28, 29]. We found that the growth of the microbial consortium on chopped straw along with 0.1% yeast extract yielded maximum enzyme activity for the degradation of both cellulose (CMC) and hemicellulose (xylan) (Fig. 3). The consortium was found to release more sugar from xylan (16.86 mg/mL) compared to CMC (0.48 mg/mL). As expected, xylan and CMC degrading activities were higher in the secreted protein fraction (Fig. 3a) as compared to cellular protein fraction (Fig. 3b).

**Fig. 4:**
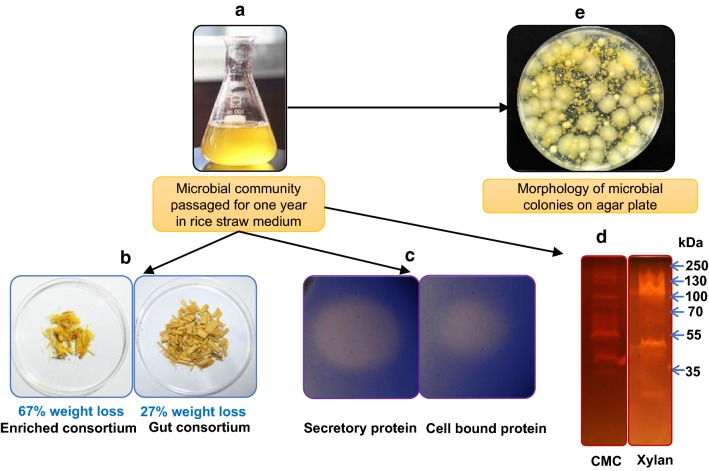
Enrichment of rice straw deconstructing YSB gut microbial community and assessment of available enzymes and biomass degrading ability. **a** The microbial community was passaged for 1 year on the rice straw containing medium and analyzed for various features. **b** Reduction in rice straw weight after incubation with either enriched consortium or original symbionts; **c** CMCase activity shown by the supernatant and cell bound protein fraction of YSB gut consortium on plate containing CMC and trypan blue dye; **d** CMCase and xylanase assay of YSB gut consortium proteins on zymogram; **e** Morphologically different colonies grown as a result of plating on YEB agar plate

The microbial consortium was subsequently sub-cultured for 1 year to facilitate enrichment and evolution of improved lignocellulolytic microbes (Fig. 4a). Significant weight reduction (67%) in the rice straw was observed after 1 week of cultivation with the enriched consortium (Fig. 4b). Culture supernatant of enriched consortium was observed for the production of enzymes with cellulolytic or/and xylanolytic activities, as indicated by the clearance zones on agar plate (Fig. 4c) and SDS-PAGE gel (Fig. 4d) containing cellulosic and hemicellulosic substrates, and showed diverse colony morphology when grown on nutrient agar plates (Fig. 4e). A separate experiment was also set up to compare the rice straw deconstruction ability of enriched YSB consortium with a non-specific gut consortium from *Spodoptera litura* (commonly known as Tobacco cutworm) (Additional file 1: Figure S1). Greater than 3.6-fold higher biomass weight reduction was observed for enriched YSB consortium as compared to gut consortium from *S. litura* (Additional file 1: Figure S1a). A similar observation was obtained when sugar release from rice straw was compared using secretome of enriched consortium with that from *S. litura* (Additional file 1: Figure S1b).

### Changes in the diversity of rice yellow stem borer gut consortium during enrichment process

16S rRNA gene analysis of the microbial community after 12 months of serial passaging on rice straw showed the enrichment of major phyla Proteobacteria and Bacteroidetes from 92.5 to 99.3%, while a decrease in relative abundance of Firmicutes and Verrucomicrobia from 7.1 to 0.2% compared to the original starting culture was observed (Fig. 1a, c). The proportion of Actinobacteria remained similar in both the gut fluid and the enriched culture at 0.3%.

There was a greater diversity of microorganisms in the original gut fluid with 178 genera identified compared to 83 in the enriched culture, and while certain strains diminished during the enrichment process others became dominant (Fig. 2a, c). For example, the top 5 genera, which constituted 65% of all genera present in the gut, were *Asticcacaulis* (37%), *Pedobacter* (11%), *Stenotrophomonas* (7%), *Rhizobium* (5%), and *Bacillus* (5%) (Fig. 2a), while in the case of the enriched culture, except for *Pedobacter* (8%), all the other genera were replaced in the top 5 ranking by *Pseudomonas* (49%), *Ensifer* (10%), *Flavobacterium* (8%), and *Aeromonas* (5%), constituting 80% of total abundance (Fig. 2c). We also observed differences between the quantitative abundance and the number of unique OTUs detected for each genus. For example, *Azotobacter* recorded the highest number of species detected under this genus in the gut consortium, while it was 7th in terms of abundance (Fig. 2a, b). In the enriched culture, *Pseudomonas* remained highest in both abundance and number of species detected, but *Azorhizophilus* was 2nd highest for number of species detected, while it was 23rd in terms of abundance (Fig. 2c, d, Additional file 1: Figure S2). More than 99.9% of genus present in enriched YSB consortium were also present in original consortium, albeit in varying abundance, suggesting that chance of contamination arising during passaging was negligible (Additional file 1: Table S1).

### Mining CAZy proteins in the enriched consortium

The enriched consortium was superior in rice straw deconstruction in liquid culture compared to the original gut microbial consortium (Fig. 4b). We, therefore, investigated the CAZy proteins produced by this enriched consortium by collecting protein samples on days 3, 7, 13, and 20 from the culture to capture proteins produced at early, mid, and late stages of the rice straw deconstruction. Metaexoproteomic analysis was performed on the secreted proteins present at each of these timepoints with a view to understanding the nature and relative abundance of potential enzymes and ancillary proteins, and also to investigate how the profile and abundance of these proteins changes over time. Secretory proteins available in two discrete fractions were extracted from the rice straw degrading cultures: a soluble extract was isolated by precipitating proteins from the culture supernatant, while a ‘bound fraction’ was obtained using a biotin-labelling methodology as described previously [30]. This methodology allowed the specific targeting of proteins tightly bound to the rice straw. Soluble and biomass-bound protein extracts were then analysed by LC–MS/MS and searched against the metatranscriptomic library generated from the enriched consortium.

**Fig. 5:**
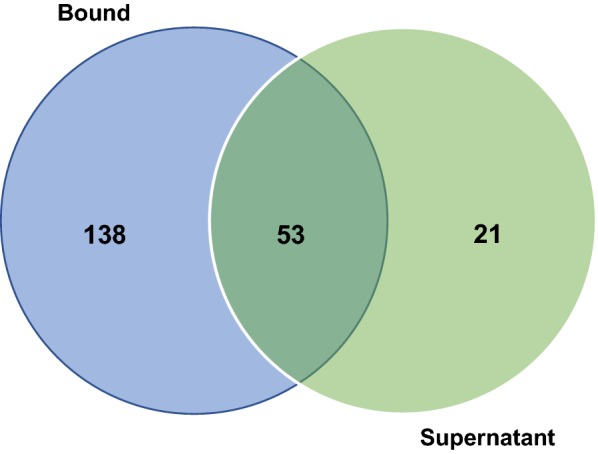
Venn diagram showing the proportion of CAZy assignments observed exclusively in the Bound Fraction, Supernatant or in both fractions

Across the four timepoints, a total of 1122 unique ORFs were identified in the YSB exoproteome, which reduced to 1088 protein hits after searching against NCBI-NR database (34 having no hits in the NR database using an *E* value cut off of 1 × 10^−5^). When these were submitted to the dbCAN database for CAZy annotation, 212 domain hits were returned (Table 2), which represented a total of 125 separate ORFs (some ORF contained more than one dbCAN domain, e.g., a GH attached to a CBM). Among those 212 CAZy domain assignments, 138 were present exclusively in the bound fraction of rice straw, 21 were exclusively present in the soluble form in the supernatant fraction, and 53 were present in both fractions (Fig. 5).

**Table 2:** CAZy families detected in rice YSB metaexoproteome

Nature of domains	Number of domains identified
Carbohydrate-binding modules (CBMs)	95
Glycoside hydrolases (GHs)	55
Carbohydrate esterases (CEs)	21
Glycosyl transferases (GTs)	19
Auxiliary activities (AAs)	16
Surface layer homology (SLH)	3
Polysaccharide lyases (PLs)	2
Dockerin	1
Total	212

Upon detailed analysis of the Glycoside Hydrolase (GH) CAZy class in the metaexoproteome, a total of 55 domains were identified that were classified into 20 GH families. Among the 55 GH domains, 51 were identified exclusively in the bound fraction (representing 19 GH families), while only one GH domain was observed exclusively in the supernatant fraction. Three GH domains from three different GH families were present in both fractions. The most abundant GH domains identified in the metaexoproteome of the enriched consortium were from families GH10, GH9, GH48, GH109, GH5, and GH6 (Table 3). When we categorized the observed GH families based on the substrate, they act upon GH48, GH6, and GH9 are known for cellulose deconstruction, GH10, GH11, GH39, and GH43 for hemicellulose deconstruction, while GH3, GH5, and GH74 are known to hydrolyze both. GH families for deconstruction of starch (GH13 and GH94), glycoproteins (GH33 and GH109) and peptidoglycans (GH20) were also identified (Table 3).

**Table 3:** Relative abundance of top 20 GH family proteins observed in the rice YSB gut consortium

Relative abundance rank	Total emPAI score^a^	Family	Bound fraction	Supernatant	Substrate
1	12.86	GH10	Yes	Yes	Hemicellulose deconstruction
2	10.67	GH9	Yes	Yes	Cellulose deconstruction
3	8.24	GH48	Yes	No	Cellulose deconstruction
4	4.38	GH109	Yes	Yes	Glycoprotein deconstruction
5	4.21	GH5	Yes	No	Cellulose and hemicellulose deconstruction
6	3.58	GH6	Yes	No	Cellulose deconstruction
7	1.28	GH74	Yes	No	Cellulose and hemicellulose deconstruction
8	1.04	GH94	Yes	No	Starch deconstruction
9	0.93	GH3	Yes	No	Cellulose and hemicellulose deconstruction
10	0.65	GH13	Yes	No	Starch deconstruction
11	0.64	GH120	Yes	No	Hemicellulose deconstruction
12	0.54	GH11	Yes	No	Hemicellulose deconstruction
13	0.37	GH15	Yes	No	Starch deconstruction
14	0.26	GH26	Yes	No	Hemicellulose deconstruction
15	0.24	GH39	Yes	No	Hemicellulose deconstruction
16	0.23	GH33	No	Yes	Glycoprotein deconstruction
17	0.12	GH43	Yes	No	Hemicellulose deconstruction
18	0.10	GH20	Yes	No	Peptidoglycan deconstruction
19	0.06	GH62	Yes	No	Hemicellulose deconstruction
20	0.03	GH2	Yes	No	Hemicellulose deconstruction

^a^Total emPAI scores are based on the sum of emPAI scores all entries for a given family of glycoside hydrolases

In terms of CBMs, a total of 95 CBMs from 15 families were identified in the enriched consortium metaexoproteome. Among those identified, 33 CBM domains (from 13 different families) were found exclusively in the bound fraction, 17 CBM domains (from 4 different families) were found exclusively in the supernatant fraction, while 45 CBM domains (representing 5 families) were identified in both fractions. By far, the most represented CBM family in the metaexoproteome was CBM44 (known for binding to cellulose and xyloglucan) accounting for 56/212 of all CAZy annotated domains. However, based on relative abundance, the most abundant CBM domain identified in the YSB metaexoproteome was CBM4 (xylan, glucan, and amorphous cellulose binding) and CBM2 (predominantly cellulose binding); their relative abundance is given in the Additional file 1: Table S2. When we categorized these CBMs on the basis of their binding specificity, we found CBM3 and CBM 63 known for cellulose binding, CBM13 and CBM22 for hemicellulose binding, while CBM2, CBM4, CBM6, CBM9, and CBM44 are known to bind both cellulose and hemicellulose. CBMs families known to bind to pectin (CBM32), starch (CBM20 and CBM48), glycoproteins (CBM32 and CBM 40), and peptidoglycans (CBM50) and chitin (CBM2 and CBM3) were also identified.

Metaexoproteome analysis also identified a total of 21 domains belonging to the Carbohydrate Esterases (CE) CAZy class and assigned to 5 families. Among them, 18 domains (representing 4 families) were present exclusively in the bound fraction, 2 domains (from 2 families) were present only in the supernatant fraction, and 1 domain was present in both. The most abundant CE domains identified in metaexoproteome were assigned to the CE1 and CE10 families; their relative abundance in each fraction is given in the Additional file 1: Table S3. In terms of substrate recognition, CE7 is known for hemicellulose deconstruction, CE1 and CE16 are known to hydrolyse hemicellulose and pectin, the CE10 domain is categorized as hemicellulose and lignin deconstructing, while the carbohydrate esterases of CE4 family have specificity for hemicellulose, chitin and peptidoglycan.

When we investigated the presence of auxiliary activities (AA) proteins in the metaexoproteome, we found a total of 16 domains designated to 3 families: AA2, AA7, and AA10. All the 16 domains were exclusively found in the bound fractions. Of all the CAZy annotated domains, the AA10 from Protein c4515_g1_i1_1 was the most abundant, and when compared with the relative abundance of all other identified proteins, it ranks 11/1088. The three AA families represented in the proteome are reported to specifically deconstruct separate components of the plant cell wall; AA10 deconstructs cellulose, AA7 deconstructs cellulose and hemicellulose, and AA2 deconstructs lignin.

In addition, the enriched consortium metaexoproteome contained polysaccharide lyases (PL) represented by two PL families: PL1 and PL2. Pectate lyase and exo-polygalacturonate lyase are two important enzymes known in these families, and they are known to depolymerise pectin present in the primary and secondary cell walls of plant biomass through eliminative cleavage.

Several proteins were found to have interesting architecture and unusual multimerization of catalytic domains or CBMs was observed in a number of ORFs (Table 4). For example, protein ID: c58415_g1_i1_1 appears to have catalytic domains of two different CAZy classes, i.e., PL and CE. Most of the multimerization was observed in the CBM44 family, where CBMs from Family 44 were repeated in the range of 2–11 (Table 4). Proteins with multimerization in auxiliary activity (AA) domain (Protein ID: c65180_g3_i1_1 and c15588_g1_i1_2, both annotated to possess three distinct AA2 domains) and carbohydrate esterases (CE) (Protein ID: c175818_g1_i1_1 annotated to have two distinct CE1 domains) have also been identified. Moreover, several proteins were identified with known CBMs, but unknown catalytic domains, for example, CBMs from families 32, 37, 40 and 44.

**Table 4:** Architecture of multi-domain CAZymes identified in the rice YSB gut consortium

S. no.	YSB contig	ORF of the YSB contig	Domain architecture of translated proteins
1	c58099_g3_i2	c58099_g3_i2_3	CBM2/CBM3/GH9
2	c65180_g3_i1	c65180_g3_i1_1	AA2/AA2/AA2
3	c66145_g1_i1	c66145_g1_i1_1	CBM20/CBM20/CBM20
4	c61378_g1_i1	c61378_g1_i1_7	CBM44/CBM44/CBM44/CBM44
5	c17840_g1_i1	c17840_g1_i1_6	CBM44/CBM44/CBM44/CBM44
6	c17840_g1_i1	c17840_g1_i1_7	CBM44/CBM44/CBM44/CBM44
7	c17840_g1_i1	c17840_g1_i1_8	CBM44/CBM44/CBM44/CBM44
8	c8173_g2_i1	c8173_g2_i1_1	CBM44/CBM44/CBM44/CBM44/CBM44
9	c66028_g1_i1	c66028_g1_i1_14	CBM44/CBM44/CBM44/CBM44/CBM44/CBM44
10	c61637_g1_i1	c61637_g1_i1_4	CBM44/CBM44/CBM44/CBM44/CBM44/CBM44/CBM44/CBM44/CBM44/CBM44/CBM44
11	c175818_g1_i1	c175818_g1_i1_1	CE1/CE1
12	c58415_g1_i1	c58415_g1_i1_1	CE1/PL22
13	c15588_g1_i1	c15588_g1_i1_2	AA2/AA2/AA2
14	c61645_g1_i2	c61645_g1_i2_15	CBM13/CBM13
15	c65434_g3_i1	c65434_g3_i1_8	CBM44/CBM44
16	c234089_g1_i1	c234089_g1_i1_4	CBM44/CBM44/CBM44/CBM44

YSB_Contigs: gene sequence obtained as a result of de novo assembly

ORF of the contigs: translated protein from different open reading frame (ORF) of the respective YSB contig

### Dynamics of CAZy protein expression

**Fig. 6:**
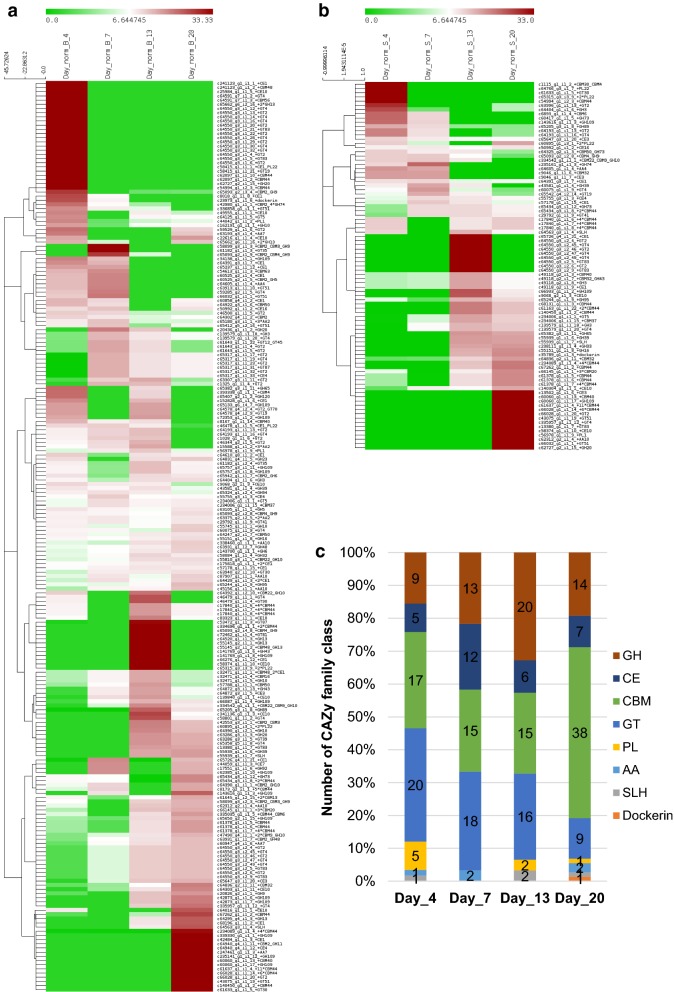
Dynamics of changes in different classes of CAZy families upon cultivation on rice straw for 20 days. Hierarchical clustering of CAZy family proteins detected at 4th, 7th, 13th and 20th day of cultivation in the bound (**a**) and supernatant (**b**) fractions. **c** Comparison of the expression level of various CAZy classes in the 30 high expressed contigs at each timepoints

The dynamics of CAZy protein expression by the enriched consortium was investigated at early, mid, and late stages of the rice straw deconstruction by performing hierarchical clustering of CAZy family proteins present at various timepoints. An ordered expression profile of CAZy family proteins was detected at various stages of cultivation both in the bound (Fig. 6a) and supernatant fractions (Fig. 6b), which indicated roles of various CAZy classes at different stages of substrate deconstruction. By comparing the expression level of various CAZy classes in the 30 highly expressed contigs at each timepoints, it appears that the number of GH family proteins increased by more than twofold in the initial stages from day 4 to day 13 (Fig. 6c). CBM numbers were more or less similar across the cultivation period, but increased by 2.5-fold mainly due to ORFs containing multi CBM44 domains. Some of the other CAZy proteins such as CE, PL, AA, SLH, and dockerins were also observed at various stages of the cultivation within the highest expressing ORFs. From the results, it appears that initially a balanced expression of key CAZy family proteins occurred, which gradually shifted towards expression of CE to de-esterify hemicellulosic sugars, followed by expression of GHs to hydrolyse the available hemicellulose and cellulose, and then the expression of a large number of CBMs to access the more recalcitrant polysaccharides.

### Recombinant expression and functional validation of a xylanase from the GH10 family

**Fig. 7:**
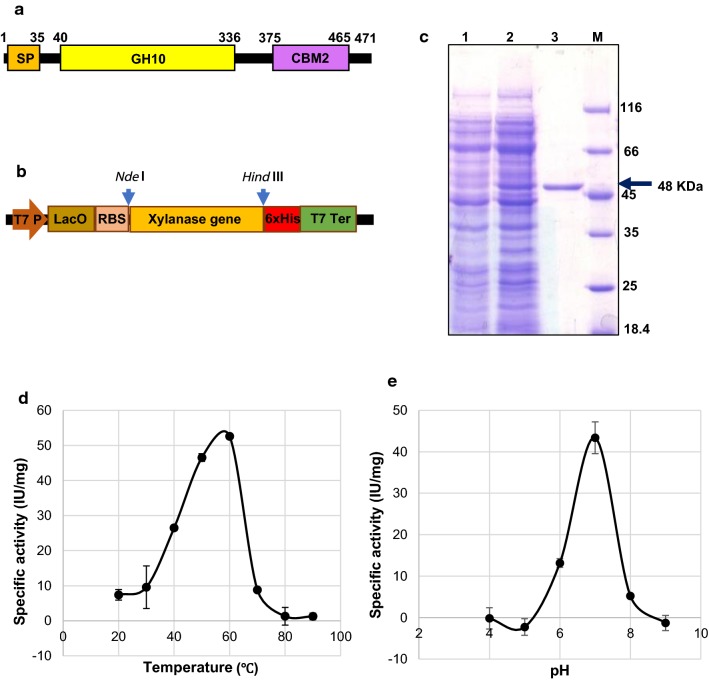
Annotation, expression and characterization of xylanase from the enriched consortium derived from rice stem borer gut. **a** Schematic representation of various modules present in the xylanase polypeptide; *SP* signal peptide, *GH10* glycoside hydrolase of family 10, *CBM2* carbohydrate-binding modules of family 2. **b** Cloning of xylanase ORF without the SP in the expression vector pET30a at the NdeI and HindIII restriction sites to derive the expression of xylanase with the help of T7 promoter. **c** Xylanase protein purification. Lane1, uninduced total cellular protein; lane 2, Induced total cellulase protein and Lane 3, Purified xylanase protein after metal affinity chromatography. **d** Optimal temperature and **e** optimal pH for activity of xylanase

A gene (Contig no. c64390_g1_i1) annotated as a xylanase belonging to CAZy GH10 family (Additional file 1: Table S4), which was in the top 10 most abundant CAZy proteins observed in the metaexoproteome, was selected for recombinant expression. The encoded protein has two CAZy domains: a GH10 catalytic domain and a CBM2 (Fig. 7a), and showed 84.13% identity at nucleotide level and 90% identity at amino acid level with *Cellulomonas* sp. Z28. The encoding gene was cloned (without the signal peptide sequence) into the expression vector pET30a (Fig. 7b) and recombinant protein expressed in *E. coli* strain shuffle (NEB), purified by metal affinity chromatography (Fig. 6c). The purified protein was active towards beechwood xylan and we found that the recombinant xylanase showed maximum activity at 60 °C, a pH optimum of 7.0 (Fig. 7d, e) and *V*_max_ and *K*_M_ values were found to be 72.2 µmol/min/mg and 2.859 mg/mL, respectively. We further assessed the biomass deconstruction ability of the recombinant enzyme and demonstrated that it was able to hydrolyze both untreated and alkali-treated rice straw. The hydrolyzate of alkali-treated rice straw contained xylobiose and xylotriose as the main products (Additional file 1: Figure S3a), while untreated rice straw only yielded xylobiose as the product (Additional file 1: Figure S3b).

## Discussion

To identify new microbial sources of lignocellulolytic enzymes, we extracted gut fluids from YSB larvae and enriched for rice straw deconstruction by sub-culturing on rice straw for over a year. As expected, we observed much higher deconstruction of rice straw by the enriched microbial consortium as compared to the freshly isolated YSB gut consortium. The enriched consortium demonstrated significant cellulase and xylanase activities and diverse colony morphology on agar plates. Since there has been little published information on the diversity of the microbiome of the rice YSB gut, we performed 16S rRNA gene analysis and explored changes in microbial population in the enriched consortium compared to the native one. The dominant species in the YSB gut consortium were Proteobacteria, Bacteroidetes, and Firmicutes, which were similar to those observed by Reetha and Mohan [31] while studying culturable microbes of the pink stem borer that is an important insect pest of several different types of crop including rice. The dominance of Proteobacteria, Bacteroidetes, and Firmicutes in the YSB gut community provides a strong indication of their importance in facilitating depolymerisation of the complex rice straw cell-wall components to monomeric sugars that can be absorbed by the host insect. Following serial sub-culturing, we observed an increase in Proteobacteria and Bacteroidetes and a decline in Firmicutes and Verrucomicrobia. As a result of cellulolytic bacteria enrichment in the consortium, we observed a decrease in the diversity of total bacterial species. Interestingly, bacterial genera known for the biomass deconstruction such as *Pseudomonas*, *Azotobacter*, *Dyadobacter*, *Flavobacterium*, *Prosthecobacter*, *Chitinophaga*, *Sphingobium*, *Pseudoxanthomonas*, *Mucilaginibacter*, *Giofilum*, *Ensifer*, and *Cellulomonas* were identified in both the original and enriched consortia.

We further cultured the enriched consortium on rice straw for 20 days and mined the CAZy proteins through metaexoproteomics. We analyzed proteins that were present in both the culture supernatant as well as those bound to the rice straw biomass [30]. Analysis of all the CAZymes present in the metaexoproteome showed that enzymes exclusively bound to the rice straw were significantly higher in abundance (9.5-fold) compared to those in the culture supernatant. In thee bound fractions, the high abundance of CAZy family proteins known for high catalytic activity on cellulose or hemicellulose such as GH10, GH9, GH48, and GH5 were identified.

In addition to single domain CAZymes, we also identified several enzymes with multi-domain molecular architecture. An enzyme was identified with a single catalytic domain and two different carbohydrate-binding modules (CBM2 and CBM3), indicating that the enzyme may possess broad specificity for different substrates. Interestingly, CAZymes with multiple repetition of CBMs belonging to families CBM13, CBM20, and CBM44, were also identified. Multimerization of CBM44 in different enzymes was in the range of 2–11 binding domains. To date, the multimerization of CBMs is mostly reported for thermostable enzymes such as CenC from *Clostridium thermocellum* [32], xylanase from *Thermoanaerobacterium aotearoense* [33], and CelA from *Caldicellulosiruptor bescii* [34]. These enzymes catalyze hydrolysis at high temperature which results in weakened binding to the insoluble substrate because of increased kinetic energy [35]. The availability of several CBMs possibly provides better accessibility of insoluble substrate to the enzyme at these higher temperatures. Moreover, some thermophilic bacteria are reported to secrete non-catalytic proteins to increase the accessibility of the insoluble substrate to the biomass deconstructing enzymes [35] and this may also apply to the consortium from the YSB. Another interesting finding is identification of several polypeptides with unknown catalytic domains linked to known CBMs. The presence of CBMs with domains of unknown function suggests that these proteins play a role in lignocellulose deconstruction and present interesting targets for characterization and for potentially boosting saccharification of biomass feedstocks.

One of the most abundant enzymes (maximum emPAI score) in the enriched consortium was a GH10 xylanase which we confirmed by showing that the recombinant enzyme was capable of hydrolyzing beechwood xylan and the hemicellulosic component of both treated and untreated rice straw.

## Conclusions

The present study was aimed at enriching a rice yellow stem borer (YSB) microbial consortium for better lignocellulosic biomass deconstruction ability, particularly against untreated rice straw. As a result, the enriched rice YSB consortium was found to deconstruct ~ 67% of the rice straw in 7 days, which is high compared to other reported microbial consortia. Wang et al. [36] found 31.5% degradation efficiency against untreated rice straw in 30 days by the rice straw adapted (RSA) compost consortia. Wongwilaiwalin et al. [37] and Yan et al. [29] reported 45% (MC3F compost consortium) and 49% (BYND-5 compost consortium) degradation efficiency against untreated rice straw in 7 days, respectively. The discovery of domains of unknown function linked to CBMs and enzymes with multi-domain architecture present interesting targets for further characterization and possible biotechnological application.

## Methods

### Rice YSB gut consortium cultivation for induced expression and mining of biomass deconstructing enzymes

The insect *Scirpophaga incertulas* commonly known as rice yellow stem borer (YSB) was selected in this study for targeted discovery of rice straw deconstructing enzymes. Insect larvae (approximately 25) were collected from the paddy fields of the Biotechnological Research Experiments field, Raipur University, Chhattisgarh, India in October 2011. Insect larvae were dissected aseptically, and the gut was isolated and microbial community harbouring in the gut was used as inoculum for further experiments. The YSB gut microbial community was inoculated in three different media: (1) Tryptic Soya Broth (TSB) (1.7% tryptone, 0.3% soya peptone, 0.25% K_2_HPO_4_, 0.5% NaCl, and 0.25% glucose); (2) rice straw in water having salt only (0.25% K_2_HPO_4_, 0.5% NaCl, and 0.5% rice straw of ~ 0.5 cm), and (3) rice straw in water having salt and 0.1% yeast extract (0.25% K_2_HPO_4_, 0.5% NaCl, 0.1% yeast extract, and 0.5% rice straw of ~ 0.5 cm). The YSB gut microbial community was cultured in three different media separately for 7 days at 30 °C with 150 rpm shaking. After 7 days, the culture was centrifuged at 10,000 rpm for 20 min, and the supernatant and cell pellet were collected separately. The supernatant was filtered through 0.22 µM syringe filter and used for enzyme assays, while the cell pellet was sonicated at 4 °C, centrifuged at 10,000 rpm and total soluble proteins (TSP) used for the enzyme assays. CMCase and xylanase assays were performed for both secretory (culture supernatant) and cell bound protein fractions collected from all three different culture and evaluated.

For enrichment of the rice straw hydrolysing microbial consortium, the insect gut microbial consortium was cultured into a medium having salt [NaCl (0.5%), K_2_HPO_4_ (0.25%)], 0.1% yeast extract, and rice straw as the main carbon source and passaged after every 7 days for 1 year. The 1 year passaged culture was evaluated for its potential biomass deconstruction ability and changes in microbial community structure or diversity.

### Enzyme assays

Enzyme assays using carboxyl methyl cellulose (CMCase) and beechwood xylan were performed as described previously [38] with some modifications. Carboxyl methyl cellulose (CMC, sigma) and beechwood xylan (HiMedia) was selected as substrate for evaluating cellulose and hemicellulose deconstruction ability of the consortium, respectively. The 250 µL of substrate (2% w/v in sodium phosphate buffer pH 7.4) was mixed with 250 µL of protein sample and incubated at 50 °C for 30 min. 500 µL of dinitrosalicylic acid (DNSA) was then added and solution was boiled at 100 °C for 5 min. The solution was cooled to room temperature and the reducing sugar content was estimated using glucose and xylose as standards for CMCase and xylanase assay, respectively. One unit of enzyme activity was defined as the amount of enzyme that released 1 μmol of reducing sugar per min.

For plate assay, an equal volume of CMC or xylan (1% w/v in water) and tryptic soya broth medium (2x) (with 1.5% agar and 0.5% trypan blue dye) was autoclaved separately. After autoclaving, both solutions were mixed together and poured into the Petri plate in laminar flow hood. The protein solution was applied on the surface of the solid agar plate under aseptic conditions and incubated at 37 °C. After 48 h, plates were visually inspected for clearance zone formation.

CMCase and xylanse activity using zymogram on SDS-PAGE gel were performed as described earlier [34]. In brief, the protein sample was resolved on a 12% SDS-PAGE gel containing either 0.5% (w/v) CMC or 0.5% (w/v) beechwood xylan. After electrophoresis, the gel was washed once with 20% (v/v) isopropanol in phosphate-buffered saline (PBS) for 1 min followed by three washes of 20 min each in PBS. The gel was incubated in PBS at 37 °C for 1 h, stained with 0.1% (w/v) Congo red for 30 min, and destained with 1 M NaCl. Clear bands against the red background indicated CMCase or xylanase activity. Protein concentrations were estimated with the bicinchoninic acid (BCA) Protein Assay kit (Pierce) using bovine serum albumin as a standard.

### Microbial diversity assessment using ion PGM sequencer platform

The original rice YSB gut consortium and the enriched consortium passaged for 1 year were processed for total DNA extraction as described in a latter section. Extracted DNA was then treated with RNase, cleaned and concentrated using Genomic DNA clean-up kit (ZymoResearch). The purified DNA was used as a template to amplify V4 hypervariable regions of the 16S rRNA gene in the consortium. Phusion High-Fidelity DNA Polymerase (Finnzymes OY, Espoo, Finland) and primer pairs covering the V4 (520 forward: 5′ AYTGGGYDTAAAGNG 3′, and 802 reverse: 5′ TACNVGGGTATCTAATCC 3′) hypervariable region [39] were used in the amplification reaction. The amplified fragments were purified with Agencourt AMPure XP (Beckman Coulter). The quantity and quality of the purified PCR products were analyzed using an Agilent Tape Station with an Agilent DNA 1000 kit. Libraries were prepared using the Ion Plus Fragment Library Kit (Life Technologies Corporation) and barcoded using Ion Xpress Barcode Adapters 1–16 Kit (Life Technologies Corporation). The libraries were quantified using Invitrogen Qubit, and an equimolar pool of initial and passaged library with unique barcodes was generated to create the final library. Template preparation was carried out with the pooled libraries using the Ion One Touch 2 system with an Ion PGM Template OT2 400 Kit (Life Technologies Corporation). Quality control at the pre-enriched template stage was made using the Ion Sphere Quality Control Kit (Life Technologies Corporation) and the Qubit 2.0 Fluorometer (Invitrogen). The templated libraries were sequenced using an Ion PGM sequencer platform (Thermo Fisher Scientific). The instrument cleaning, initialization, and sequencing was done by reagents provided in the Ion PGM 400 Sequencing Kit (Life Technologies Corporation) using an Ion314 Chip v2.

### Data processing and analysis for microbial diversity

Amplicon Fastq files were converted to Fasta and quality files using QIIME convert_fastaqual_fastq.py script [40]. The resulting files were quality filtered by removing reads outside the minimum (− l 180) and maximum (− *L* 250) read length and quality score (*Q* < 25). During the split_libraries.py process, forward and reverse primer sequences were also trimmed. Filtered files were concatenated and replicated sequences with a minimum size of two were removed with VSEARCH-derep_fulllength command [41]. OTU clustering and chimera filtering were performed using UPARSE–cluster_otu command [42] at 97% identity. The pipeline produced two output files, an OTU table in txt format (further converted into biom file format), and a set of representative sequences for each OTU in fasta format. The representative sequences were then assigned to taxonomy using UCLUST [43] and Greengenes database [44] as a reference on QIIME (assign_taxonomy.py). Taxonomy was added to the OTU table using biom add-metadata script. Running a default command on QIIME, alpha and beta diversity and taxonomy summary analyses were performed. Visualization and statistical analysis was done using Prism7.

### Experimental design and sample collection for metatranscriptomic and metaexoproteomic study

To investigate candidate biomass deconstructing proteins/enzymes and their encoding genes, metaexoproteomics and metatranscriptomics of the stable rice YSB gut consortium were performed, respectively. Three replicates of 2 L flasks containing 500 mL medium (0.5% NaCl, 0.25% K_2_HPO_4_, 1% Yeast Extract, pH 7) with 1.5% rice straw were prepared and autoclaved, and 2% YSB seed culture was inoculated, cultured by incubating at 30 °C and 150 rpm for 20 days. In addition to these three cultures, a negative control flask was also set up as outlines above, but without the addition of the YSB seed culture. 100 mL samples were collected at 3, 7, 13, and 20 day post-inoculation for protein and DNA/RNA extraction for metaexoproteomics and metatranscriptomics, respectively.

### DNA and RNA extraction

Triplicate samples of DNA and RNA were extracted from all three cultures and the negative at each timepoints by following the protocol reported previously [45] with some modification. In brief, collected samples were spun at 12,000×*g* at 4 °C for 10 min. Supernatant was used for protein preparation, while pelleted biomass (microbial and rice straw) was used for DNA/RNA preparation. 0.5 g of the biomass pellet was transferred into 2 mL microcentrifuge tube containing glass beads (0.5 g, 0.5 mm and 0.5 g, 0.1 mm), and 0.5 mL CTAB buffer (10% CTAB in 0.7 M NaCl, 240 mM potassium phosphate buffer, pH 8.0, and 1 µL β-mercaptoethanol/mL buffer) was added and vortexed. For nucleic acid extraction, 0.5 mL phenol:chloroform:isoamyl alcohol (25:24:1, pH 8.0) was added, mixed, and then homogenised using a TissueLyser II (Qiagen) for 4 × 2.5 min at a speed setting of 28 s^−1^. The samples were phase separated by centrifugation at 13,000×*g*, 4 °C for 10 min, and the resulting aqueous phase was extracted with an equal volume of chloroform:isoamyl alcohol (24:1). The nucleic acids were precipitated overnight at 4 °C from the final aqueous fraction by adding 2 volumes of precipitation solution (1.6 M NaCl, 20% PEG8000 buffer 0.1% DEPC treated). The resulting pellet was washed twice with 1 mL ice-cold 75% ethanol, air-dried, and re-suspended in 50 μL RNase/DNase free water.

### Metatranscriptome (Illumina shotgun) sequencing

A sample of the extracted nucleic acids was treated to remove DNA by addition of DNase (Mo Bio, USA) as recommended by manufacturers. Total RNA was then processed for small RNA removal and purification by RNA Clean and Concentrator kit (Zymo Research, USA). For each timepoint purified total RNA (0.7 µg) from all three biological replicates were pooled (total 2.1 µg) and processed for ribosomal RNA removal using Ribo-Zero™ Magnetic Gold (Epidemiology) kit (Epicentre or Illumina, USA), using the protocol recommended by manufacturer. The quality of ribosomal RNA (rRNA)-depleted sample was analyzed using an Agilent TapeStation 2200 using High Sensitivity (HS) RNA ScreenTape (Agilent, USA). Finally, 100 ng rRNA depleted RNA was used for library preparation to perform sequencing on Illumina 2500 platform (Illumina, USA). For all four timepoints the library was prepared using TruSeq RNA Sample Prep v2 kit (Part# 15026495, Illumina) and the protocol was adapted as recommended by the manufacturer. During library preparation different indexing adapters were added to the pooled RNA samples for each of the four timepoints. These four libraries were normalized with equimolar amounts of each library, pooled and subsequently diluted to 10 pM.

For sequencing, rapid run mode was followed. The library template along with 1% PhiX template hybridised onto an Illumina flow cell (single lane) placed on cBot system, and complete cluster generation was done on the HiSeq 2500 instrument. TruSeq Rapid PE Clusture v1 kit (Illumina) was used for cluster generation following the protocol recommended by the manufacturer. Sequencing by synthesis (SBS) chemistry was applied for clustered library sequencing using TruSeq Rapid SBS v1 kit for 100 cycles for each pair end reads. HiSeq Control Software (HCS) 2.2.58, Real-Time Analysis software 1.18.64 and Sequencing analysis viewer software was used in sequencing run processing and data acquisition. Sequences were obtained in the form of reads in BCL format. Reads were demultiplexed by removing 6 bp index using the CASAVA v1.8 program allowing for a one base-pair mismatch per library, and converted to FASTQ format using bcl2fastq. The sequenced libraries were searched against SILVA 115 database [46] to identify rRNA genes using Bowtie 2 software [47]. Those reads as well as orphans and poor quality sequences were removed with the next-generation sequencing Short Reads Trimmer (ngsShoRT) software. Filtered reads from all timepoints were pooled prior to assembly, the Trinity package [48] with a k-mer length of 43 was used for de novo assembly.

### Metaexoproteomics of enriched gut consortium

A sample of the biomass deconstructing enriched microbial community culture (30 mL) was collected at all four timepoints from all three biological replicates. This was centrifuged at 12,000×*g* at 4 °C for 10 min. Both supernatant and pelleted biomass fractions were collected to be processed for protein concentration and LC–MS/MS analysis. The 3 × 5 mL of the collected supernatant was precipitated by addition of 100% ice-cold acetone after filtering it through 0.22 µm syringe filter, and incubated for 16 h at − 20 °C. The precipitated protein was collected by centrifugation at 10,000×*g* and washed two times with 80% ice-cold acetone. Pellets were finally air-dried and re-suspended in 0.5 × phosphate buffer saline (PBS, 68 mM NaCl, 1.34 mM KCl, 5 mM Na_2_HPO_4_, 0.88 mM KH_2_PO_4_), snap frozen and stored at − 80 °C till processed for next step.

The pelleted biomass fraction was presumed to contain microbes, rice straw and secreted proteins attached to both. In triplicate, 2 g of biomass were aliquoted into 50 mL tubes and washed twice with 25 mL ice-cold 0.5× PBS buffer. Washed biomass was re-suspended in 19 mL 0.5× PBS, with the addition of 10 mM freshly prepared EZ-link-Sulfo-NHS-SS-biotin (Thermo Scientific) and incubated with rotator at 4 °C for 1 h. Samples were pelleted (10,000×*g*, at 4 °C for 10 min), and the supernatant discarded. The biotinylated reaction was quenched by the addition of 25 mL 50 mM Tris–Cl pH 8.0 and a further 30 min incubation with rotation at 4 °C. The soluble fraction was recovered and washed twice with 0.5× PBS, and bound proteins liberated by resuspension in 10 mL of 2% SDS (pre-heated to 60 °C), incubated at room temperature for 1 h with rotation. To recover the liberated biotin-labelled proteins, the samples were clarified by centrifugation (10,000×*g*, 4 °C for 10 min) and the supernatant was collected. The protein present in supernatant was precipitated with ice-cold acetone and incubated at − 20 °C for 16 h. Precipitate was then washed twice with 80% ice-cold acetone, air-dried and re-suspended in 1 mL 1× PBS containing 0.1% SDS. Re-suspended proteins were filtered through 0.2 µm filter and loaded onto a HiTrap™ Streptavidin HP column (GE, Sweden) pre-packed with 1 mL Streptavidin immobilized on a Sepharose beads matrix. The column was equilibrated with 10 column volume (CV) PBS containing 0.1% SDS (equilibration buffer). After protein loading column was washed with 10 column volumes (CV) 1× PBS containing 0.1% SDS (equilibration buffer). For elution of bound protein, freshly prepared 1 mL of 1× PBS buffer containing 50 mM DTT (elution buffer) was added into the column and incubated overnight at 4 °C before eluting.

In preparation of label-free LC–MS/MS, both bound fraction proteins and samples of protein collection from culture supernatant were desalted using 7 k MWCO Zeba Spin desalting column (ThermoFisher scientific, USA) according to the manufacturer instructions. Protein samples were then freeze dried and re-suspended in SDS-PAGE protein loading buffer, loaded onto 10% Bis–Tris gels and resolved for 6 min at 180 V to store protein samples in-gel. After staining, protein bands were excised and stored at − 80 °C prior to LC–MS/MS analysis.

### Liquid chromatography coupled tandem mass spectrometric analysis

The sliced gel pieces were subjected to tryptic digestion after reduction and alkylation. The resulting peptides were reconstituted in 0.1% trifluoroacetic acid (TFA) and processed for nano LC–MS/MS as described previously [49]. In brief, reconstituted peptides were loaded onto a nanoAcquity UPLC system (Waters, Milford, MA, USA) equipped with a nanoAcquity Symmetry C18, 5-μm trap (180 μm × 20 mm) and a nanoAcquity BEH130 1.7-μm C18 capillary column (75 μm × 250 mm). The trap was washed for 5 min with 0.1% aqueous formic acid having flow rate of 10 μL/min before switching flow to the capillary column. Separation on the capillary column was achieved by gradient elution of two solvents (solvent A: 0.1% formic acid in water; solvent B: 0.1% formic acid in acetonitrile) with a flow rate of 300 nL/min. The column temperature was 60 °C, and the gradient profile was as follows: initial conditions 5% solvent B (2 min), followed by a linear gradient to 35% solvent B over 20 min and then a wash with 95% solvent B for 2.5 min. The nanoLC system was interfaced with a maXis liquid chromatography coupled to tandem mass spectrometry (LC-Q-TOF) system (Bruker Daltonics) with a nanoelectrospray source fitted with a steel emitter needle (180 μm o.d. × 30 μm i.d.; roxeon). Positive electron spray ionization (ESI)-MS and MS/MS spectra were acquired using AutoMSMS mode. Instrument control, data acquisition, and processing were performed using Compass 1.3 SP1 software (microTOF control HyStar, and Data Analysis software; Bruker Daltonics). The following instrument settings were used: ion spray voltage = 1400 V; dry gas 4 L/min; dry gas temperature = 160 °C and ion acquisition range *m/z* 50–2200. AutoMSMS settings were as follows: MS = 0.5 s (acquisition of survey spectrum); MS/MS [collision induced dissociation (CID) with N2 as collision gas]; ion acquisition range, *m/z* = 350–1400; 0.1-s acquisition for precursor intensities above 100,000 counts; for signals of lower intensities down to 1000 counts acquisition time increased linear to 1.5 s; the collision energy and isolation width settings were automatically calculated using the AutoMSMS fragmentation table; 3 precursor ions, absolute threshold 1000 counts, preferred charge states, 2–4; singly charged ions excluded. Two MS/MS spectra were acquired for each precursor and former target ions were excluded for 60 s.

Acquired data from MS/MS was searched against the previously prepared YSB metatranscriptome data base using Mascot search engine (Matrix Science Ltd., version 2.4) through the Bruker ProteinScape interface version 2.1). The following parameters were applied: tryptic digestion, carbamidomethyl cysteine as fixed modification, oxidized methionine and deamidation of asparagine and glutamine as the variable modification. A maximum of one missed cleavages were allowed. The peptide mass tolerance was set to 10 ppm and MS/MS fragment mass tolerance was set to 0.1 Da. Protein false discovery rate (FDR) was adjusted to 1%. A minimum of two significant peptides and one unique peptide were required for each identified protein.

### Bioinformatic analysis of metaexoproteomes

Nucleotide sequences of contigs matching to observed proteins by Mascot were retrieved from the metatranscriptomic databases using Blast-2.2.30 + Standalone. EMBOSS [50] application was used to generate all possible open reading frames (ORFs) from these matched contigs, defined as any region > 300 bases between a start (ATG) and a stop codon. These ORF libraries were converted into amino acid sequences and these proteins were annotated using BLASTP searching against the non-redundant NCBI database with an *E* value threshold of 1 × 10^−5^. Protein sequences were also annotated using dbCAN [51] to identify likely carbohydrate-active domains. Subcellular localisation was predicted using SignalP v. 4.1 [52] program with the default cut off value.

### Functional validation of rice YSB gut symbionts’ xylanase affiliated to family GH10

Open reading frame (1416 bp) of the metatranscriptome assembled contig no. c64390_g1_i1 encoding putative endoxylanase of CAZy family GH10 was selected for functional validation in *Escherichia coli*. The encoded protein was 471 amino acids including an N-terminal signal peptide of 35 amino acids. For recombinant expression, the encoding gene without signal peptide of 1320 bp was codon optimized and synthesised commercially (Genscript), and subcloned in pET30a vector at NdeI and HindIII sites. This construct was transformed into BL21(DE3) and SHuffle (NEB) strain of *E. coli*. Expression profiles for both the expression hosts were evaluated on SDS-PAGE and due to higher expression levels of target soluble protein in SHuffle cells, these cells were selected for scaled up protein expression in 2 litre culture, followed by affinity purification of recombinant xylanase using Ni–NTA agarose matrix (Qiagen). Concentration of the purified protein was determined using BCA Protein Assay kit as described earlier.

The enzymatic activity of the purified protein was tested for its ability to hydrolyse CMC (carboxy methyl cellulose, Sigma), PASC (phosphoric acid swollen cellulose prepared from Avicel pH 101, Sigma) and Xylan (Beechwood Xylan, HiMedia). The released reducing sugars were measure when the recombinant protein was incubated with number of different substrate by the dinitrosalicylic acid (DNSA) method as described previously [53]. Briefly, a crude enzyme solution (0.125 mL) was mixed with 0.125 mL of a 2% substrate solution in 20 mM Tris–Cl pH 7.0 buffer and incubated at 50 °C for 30 min. Enzymatic reactions against PASC was incubated for 60 min. The reducing sugar produced in these experiments was measured by the DNS reagent at 540 nm. One unit of enzymatic activity was defined as the amount of enzyme that released 1 µmol of reducing sugar from the substrate per minute under the above conditions.

### Determination of optimal reaction conditions, kinetic parameters and biomass hydrolysis capability of recombinant RSB_GH10_Xylanase

The optimum temperature for maximum xylanase activity was determined by varying the enzymatic reaction temperature in the range of 40–100 °C. For optimum pH assessment, purified protein was dialysed against buffers ranging in pH from 4 to 9. The buffer for pH range 4–6 was 20 mM Citrate buffer containing 150 mM NaCl, while buffer for pH range 7–9 was 20 mM Tris–Cl contacting 150 mM NaCl. Activity assays were performed as described previously.

The kinetic parameters of recombinant xylanase were determined using beechwood xylan with substrate concentrations ranging from 0.5 to 10 mg/mL in 20 mM phosphate buffer (pH 7.0) at 60 °C. The kinetic constants, K_M_ and Vmax, were estimated using GraphPad Prism 7.02 (GraphPad Sofware, Inc., San Diego, CA).

Rice straw deconstruction by recombinant RSB_GH10_Xylanase was determined as follows. Sodium hydroxide treated and untreated rice straw (kindly provided by Prof. Arvind Lali) were deconstructed by incubating 16 mg with purified 30 µg recombinant xylanase for 8 h at 60 °C. After incubation, the reaction mixture was centrifuged at 20,000×*g* for 15 min, supernatant was filtered through 0.45 µm filter and analyzed on Aminex column (Bio-Rad) using xylotetrose, xylotriose, xylobiose and xylose as standards. Biomass incubated with buffer and protein incubated with buffer were used as used as negative controls.

## Supplementary information

**Additional file 1: Table S1.** Relative abundance of microbes in the enriched consortium compared to the original consortium. **Table S2** Carbohydrate-Binding Modules (CBM) family proteins observed in the rice YSB gut consortium. **Table S3** Carbohydrate Esterases (CE) family proteins observed in the rice YSB gut consortium. **Table S4** Relative ranking of top 18 CAZy family proteins of different classes as observed in the rice YSB gut consortium based on emPAI score. **Figure S1** (a) Reduction in rice straw weight after 7 days of incubation with different gut consortium, with uninoculated medium as a control. (b) Glucose release after incubation of supernatant of different consortium with rice straw for 7 days. **Figure S2** Change in community structure as a result of enrichment**. Figure S3** Alkali-treated (a) and untreated (b) rice straw hydrolysis and product analysis.

## Data Availability

All data supporting the conclusions of this article are included within the manuscript and in the additional information.

## Abbreviations

YSB: yellow stem borer
MT: metatranscriptome
LC–MS/MS: liquid chromatography–tandem mass spectrometry
OTU: operational taxonomic unit
COG: cluster of orthologous group
CAZymes: carbohydrate‑active enzymes
GH: glycosyl hydrolase
AA: auxiliary activities
CBM: carbohydrate‑binding module
GT: glycosyl transferase
PL: polysaccharide lyase
ORF: open reading frame
Xyl: xylanase

## References

[1] Saini JK, Saini R, Tewari L (2015). Lignocellulosic agriculture wastes as biomass feedstocks for second-generation bioethanol production: concepts and recent developments. 3 Biotech.

[2] Sarkar N, Ghosh SK, Bannerjee S, Aikat K (2012). Bioethanol production from agricultural wastes: an overview. Renew Energy.

[3] Lee J (1997). Biological conversion of lignocellulosic biomass to ethanol. J Biotechnol.

[4] Prasad S, Singh A, Joshi HC (2007). Ethanol as an alternative fuel from agricultural, industrial and urban residues. Resour Conserv Recycl.

[5] Anwar Z, Gulfraz M, Irshad M (2014). Agro-industrial lignocellulosic biomass a key to unlock the future bio-energy: a brief review. J Radiat Res Appl Sci..

[6] Abbas A, Ansumali S (2010). Global potential of rice husk as a renewable feedstock for ethanol biofuel production. Bioenergy Res..

[7] Binod P, Sindhu R, Singhania RR, Vikram S, Devi L, Nagalakshmi S (2010). Bioethanol production from rice straw: an overview. Bioresour Technol.

[8] Iqbal HM, Kyazze G, Keshavarz T (2013). Advances in the valorization of lignocellulosic materials by biotechnology: an overview. BioResources.

[9] Satlewal A, Agrawal R, Bhagia S, Das P, Ragauskas AJ (2018). Rice straw as a feedstock for biofuels: availability, recalcitrance, and chemical properties. Biofuels Bioprod Biorefin.

[10] Han YW, Anderson AW (1974). The problem of rice straw waste a possible feed through fermentation. Econ Bot.

[11] Drake D, Nader G, Forero L. Feeding rice straw to cattle. UCANR Publications; 2002.

[12] Gadde B, Bonnet S, Menke C, Garivait S (2009). Air pollutant emissions from rice straw open field burning in India, Thailand and the Philippines. Environ Pollut..

[13] Mittal SK, Singh N, Agarwal R, Awasthi A, Gupta PK (2009). Ambient air quality during wheat and rice crop stubble burning episodes in Patiala. Atmos Environ.

[14] Satyendra T, Singh RN, Shaishav S (2013). Emissions from crop/biomass residue burning risk to atmospheric quality. Int Res J Earth Sci..

[15] Elkins JG, Raman B, Keller M (2010). Engineered microbial systems for enhanced conversion of lignocellulosic biomass. Curr Opin Biotechnol.

[16] Lynd LR, Laser MS, Bransby D, Dale BE, Davison B, Hamilton R (2008). How biotech can transform biofuels. Nature Biotechnol..

[17] Shi W, Ding SY, Yuan JS (2011). Comparison of insect gut cellulase and xylanase activity across different insect species with distinct food sources. Bioenergy Res..

[18] Krishnan M, Bharathiraja C, Pandiarajan J, Prasanna VA, Rajendhran J, Gunasekaran P (2014). Insect gut microbiome—an unexploited reserve for biotechnological application. Asian Pac J Trop Biomed..

[19] Meyer AS, Rosgaard L, Sørensen HR (2009). The minimal enzyme cocktail concept for biomass processing. J Cereal Sci.

[20] Willis JD, Oppert C, Jurat-Fuentes JL (2010). Methods for discovery and characterization of cellulolytic enzymes from insects. Insect Sci.

[21] Lynd LR (1996). Overview and evaluation of fuel ethanol from cellulosic biomass: technology, economics, the environment, and policy. Ann Rev Energy Env..

[22] Pathak MD, Khan ZR (1994). Insect pests of rice.

[23] Rubin EM (2008). Genomics of cellulosic biofuels. Nature.

[24] Watanabe H, Tokuda G (2010). Cellulolytic systems in insects. Annu Rev Entomol.

[25] Fischer R, Ostafe R, Twyman RM (2013). Cellulases from insects. Yellow Biotechnology.

[26] Gomez LD, Steele-King CG, McQueen-Mason SJ (2008). Sustainable liquid biofuels from biomass: the writing’s on the walls. New Phytol.

[27] Ghio S, Insani EM, Piccinni FE, Talia PM, Grasso DH, Campos E (2016). GH10 XynA is the main xylanase identified in the crude enzymatic extract of *Paenibacillus* sp. A59 when grown on xylan or lignocellulosic biomass. Microbiol Res..

[28] Wang W, Yan L, Cui Z, Gao Y, Wang Y, Jing R (2011). Characterization of a microbial consortium capable of degrading lignocellulose. Bioresour Technol.

[29] Yan L, Gao Y, Wang Y, Liu Q, Sun Z, Fu B (2012). Diversity of a mesophilic lignocellulolytic microbial consortium which is useful for enhancement of biogas production. Bioresour Technol.

[30] Alessi AM, Bird SM, Oates NC, Li Y, Dowle AA, Novotny EH, Bennett JP, Polikarpov I, Young JP, McQueen-Mason SJ, Bruce NC (2018). Defining functional diversity for lignocellulose degradation in a microbial community using multi-omics studies. Biotechnol Biofuels.

[31] Reetha BM, Mohan M (2018). Diversity of commensal bacteria from mid-gut of pink stem borer (*Sesamia inferens* [Walker])-Lepidoptera insect populations of India. J Asia Pac Entomol..

[32] ul Haq I, Akram F, Khan MA, Hussain Z, Nawaz A, Iqbal K, Shah AJ (2015). CenC, a multidomain thermostable GH9 processive endoglucanase from *Clostridium thermocellum*: cloning, characterization and saccharification studies. World J Microbiol Biotechnol..

[33] Huang X, Li Z, Du C, Wang J, Li S (2015). Improved expression and characterization of a multidomain xylanase from *Thermoanaerobacterium aotearoense* SCUT27 in *Bacillus subtilis*. J Agric Food Chem.

[34] Yi Z, Su X, Revindran V, Mackie RI, Cann I (2013). Molecular and biochemical analyses of CbCel9A/Cel48A, a highly secreted multi-modular cellulase by *Caldicellulosiruptor bescii* during growth on crystalline cellulose. PLoS ONE.

[35] Yokoyama H, Yamashita T, Morioka R, Ohmori H (2014). Extracellular secretion of noncatalytic plant cell wall-binding proteins by the cellulolytic thermophile *Caldicellulosiruptor bescii*. J Bacteriol.

[36] Wang C, Dong D, Wang H, Müller K, Qin Y, Wang H (2016). Metagenomic analysis of microbial consortia enriched from compost: new insights into the role of Actinobacteria in lignocellulose decomposition. Biotechnol Biofuels.

[37] Wongwilaiwalin S, Rattanachomsri U, Laothanachareon T, Eurwilaichitr L, Igarashi Y, Champreda V (2010). Analysis of a thermophilic lignocellulose degrading microbial consortium and multi-species lignocellulolytic enzyme system. Enzyme Microb Technol.

[38] Bashir Z, Kondapalli VK, Adlakha N, Sharma A, Bhatnagar RK, Chandel G (2013). Diversity and functional significance of cellulolytic microbes living in termite, pill-bug and stem-borer guts. Sci Rep..

[39] Klindworth A, Pruesse E, Schweer T, Peplies J, Quast C, Horn M, Glöckner FO (2013). Evaluation of general 16S ribosomal RNA gene PCR primers for classical and next-generation sequencing-based diversity studies. Nucleic Acids Res.

[40] Caporaso JG, Kuczynski J, Stombaugh J, Bittinger K, Bushman FD, Costello EK (2010). QIIME allows analysis of high-throughput community sequencing data. Nat Methods.

[41] Rognes T, Flouri T, Nichols B, Quince C, Mahé F (2016). VSEARCH: a versatile open source tool for metagenomics. Peer J..

[42] Edgar RC (2013). UPARSE: highly accurate OTU sequences from microbial amplicon reads. Nat Methods.

[43] Edgar RC (2010). Search and clustering orders of magnitude faster than BLAST. Bioinformatics.

[44] McDonald D, Price MN, Goodrich J, Nawrocki EP, DeSantis TZ, Probst A (2012). An improved Greengenes taxonomy with explicit ranks for ecological and evolutionary analyses of bacteria and archaea. ISME J.

[45] Griffiths RI, Whiteley AS, O’Donnell AG, Bailey MJ (2000). Rapid method for coextraction of DNA and RNA from natural environments for analysis of ribosomal DNA-and rRNA-based microbial community composition. Appl Environ Microbiol.

[46] Quast C, Pruesse E, Yilmaz P, Gerken J, Schweer T, Yarza P, Peplies J, Glöckner FO (2013). The SILVA ribosomal RNA gene database project: improved data processing and web-based tools. Nucleic Acids Res.

[47] Langmead B, Salzberg SL (2012). Fast gapped-read alignment with Bowtie 2. Nat Methods.

[48] Grabherr MG, Haas BJ, Yassour M, Levin JZ, Thompson DA, Amit I (2011). Full-length transcriptome assembly from RNA-Seq data without a reference genome. Nat Biotechnol.

[49] Kern M, McGeehan JE, Streeter SD, Martin RN, Besser K, Elias L, Eborall W, Malyon GP, Payne CM, Himmel ME, Schnorr K (2013). Structural characterization of a unique marine animal family 7 cellobiohydrolase suggests a mechanism of cellulase salt tolerance. Proc Natl Acad Sci USA.

[50] Rice P, Longden I, Bleasby A (2000). EMBOSS: the European molecular biology open software suite. Trends Genet.

[51] Yin Y, Mao X, Yang J, Chen X, Mao F, Xu Y (2012). dbCAN: a web resource for automated carbohydrate-active enzyme annotation. Nucleic Acids Res.

[52] Nielsen H, Engelbrecht J, Brunak S, Von Heijne G (1997). Identification of prokaryotic and eukaryotic signal peptides and prediction of their cleavage sites. Protein Eng.

[53] Adlakha N, Rajagopal R, Kumar S, Reddy VS, Yazdani SS (2011). Synthesis and characterization of chimeric proteins based on cellulase and xylanase from an insect gut bacterium. Appl Environ Microbiol.

