# Supplementary material for: Mining the biomass deconstructing capabilities of rice yellow stem borer symbionts
Source: Biotechnol Biofuels. 2019 Nov 8;12:265. doi: 10.1186/s13068-019-1603-8 (PMC6839054; doi:10.1186/s13068-019-1603-8)
Supplement: Supplementary file 1 — Additional file 1: Table S1. Relative abundance of microbes in the enriched consortium compared to the original consortium. Table S2 Carbohydrate-Binding Modules (CBM) family proteins observed in the rice YSB gut consortium. Table S3 Carbohydrate Esterases (CE) family proteins observed in the rice YSB gut consortium. Table S4 Relative ranking of top 18 CAZy family proteins of different classes as observed in the rice YSB gut consortium based on emPAI score. Figure S1 (a) Reduction in rice straw weight after 7 days of incubation with different gut consortium, with uninoculated medium as a control. (b) Glucose release after incubation of supernatant of different consortium with rice straw for 7 days. Figure S2 Change in community structure as a result of enrichment. Figure S3 Alkali-treated (a) and untreated (b) rice straw hydrolysis and product analysis. [file 13068_2019_1603_MOESM1_ESM.pdf]

Methods for Additional File

Rice straw deconstruction ability of rice YSB gut consortium cultured into the optimised medium with 0.5% rice straw was compared with gut consortium of *Spodoptera litura* (commonly known as Tobacco cutworm (CW)). Tobacco cutworm larvae collected from insectary of National Institute of Plant Genome Research, New Delhi, and gut consortium isolated and cultured in similar conditions as described for rice YSB gut consortium, with uninoculated culture medium used as a control. For comparison study, equal microbial cell density ($2.4*10^6$ cells/ml) of gut microbes of *Spodoptera litura* larvae and enriched and un-enriched gut microbe of *Scirpophaga incertulas* larvae were inoculated, along with controls in flask containing 20 ml 1% rice straw containing media for 7 days at 30 °C and 150 rpm. After 7 days, culture were pelleted, dried and percentage weight reduction in rice straw was calculated (Figure S1a). The supernatants of these cultures were further incubated with 1% rice straw at 50 °C for 7 days and glucose release was estimated by HPLC (Figure S1b).

Table S1. Relative abundance of microbes in enriched consortium compared to original consortium

S. No.	Genus in Enriched Consortium	Relative abundance in Enriched Consortium	Relative abundance in Original Consortium
**1**	*Pseudomonas*	49.304	2.985
**2**	*Ensifer*	9.797	0.389
**3**	*Flavobacterium*	8.426	0.881
**4**	*Pedobacter*	7.502	11.076
**5**	*Aeromonas*	5.016	2.423
**6**	*Mucilaginibacter*	2.920	0.180
**7**	*Geofilum*	2.776	0.163
**8**	*Pseudoxanthomonas*	2.476	0.473
**9**	*Stenotrophomonas*	2.338	7.424
**10**	*Azotobacter*	1.418	2.467
**11**	*Ancylobacter*	1.271	0.061
**12**	*Shinella*	0.910	0.348
**13**	*Flavihumibacter*	0.862	0.022
**14**	*Rhizobium*	0.772	5.033
**15**	*Phenylobacterium*	0.571	0.020
**16**	*Terrimonas*	0.568	0.072
**17**	*Achromobacter*	0.542	0.132
**18**	*Sphingobium*	0.318	0.511
**19**	*Zavarzinella*	0.262	0.004
**20**	*Bosea*	0.222	0.622
**21**	*Cellulomonas*	0.216	0.184
**22**	*Hydrogenophaga*	0.204	0.059
**23**	*Azorhizophilus*	0.188	0.000
**24**	*Sphingopyxis*	0.177	0.002
**25**	*Viridibacillus*	0.154	0.782
**26**	*Devosia*	0.153	0.066
**27**	*Roseomonas*	0.116	0.232
**28**	*Mycoplana*	0.082	0.026
**29**	*Rhizorhabdus*	0.065	0.059
**30**	*Asticcacaulis*	0.051	36.780
**31**	*Chitinophaga*	0.043	1.195
**32**	*Chelatococcus*	0.043	0.043
**33**	*Ramlibacter*	0.037	0.897
**34**	*Mycetocola*	0.034	0.045
**35**	*Mesorhizobium*	0.031	0.003
**36**	*Azospirillum*	0.020	0.708
**37**	*Asinibacterium*	0.017	0.269
**38**	*Bdellovibrio*	0.015	0.055

**39**	*Nubsella*	0.009	0.013
**40**	*Saccharibacillus*	0.008	0.001
	**Total**	**99.932**	**76.704**

Table S2 Carbohydrate Binding Modules (CBM) family proteins observed in the rice YSB gut consortium

Relative abundance rank	Total emPAI score#	Family	Bound fraction	Supernatant	Substrate
1	13.11	CBM2	Y	N	Cellulose, hemicellulose and chitin
2	9.72	CBM44	Y	Y	Cellulose and hemicellulose
3	7.86	CBM4	Y	N	Cellulose and hemicellulose
4	3.03	CBM20	Y	Y	Starch
5	2.40	CBM3	Y	N	Cellulose and chitin
6	2.10	CBM13	Y	N	Hemicellulose
7	1.80	CBM9	Y	Y	Cellulose and hemicellulose
8	1.60	CBM22	Y	Y	Hemicellulose
9	0.88	CBM50	Y	N	Peptidoglycan
10	0.41	CBM48	Y	Y	Starch
11	0.26	CBM23	Y	N	Mannan
12	0.24	CBM32	N	Y	Pectin and glycoprotein
13	0.24	CBM63	Y	N	Cellulose
14	0.16	CBM6	Y	N	Cellulose and hemicellulose
15	0.03	CBM40	Y	N	Glycoprotein

Total emPAI scores are based on the sum of emPAI scores all entries for a given family of glycoside hydrolases

Table S3 Carbohydrate Esterases (CE) family proteins observed in the rice YSB gut consortium

Relative abundance rank	Total emPAI score#	Family	Bound fraction	Supernatant	Substrate
1	16.47	CE1	Y	Y	Hemicellulose and pectin
2	2.19	CE10	Y	N	Hemicellulose and lignin
3	0.55	CE16	Y	Y	Hemicellulose and pectin
4	0.09	CE7	Y	N	Hemicellulose
5	0.09	CE4	Y	N	Hemicellulose, chitin and peptidoglycan

Total emPAI scores are based on the sum of emPAI scores all entries for a given family of glycoside hydrolases

Table S4 Relative ranking of top 18 CAZy family proteins of different classes as observed in the rice YSB gut consortium based on emPAI score

CAZy rank#	Contig ID	Family	emPAI score	Interesting target information
**1**	c45156_g1_i1_1	AA10	11.14	Most abundant CAZY protein, annotated as a lytic polysaccharide monooxygenase
**2**	c175818_g1_i1_1	CE1	6.61	Annotated as 2x CE1 domains - esterases acting on xylan & lignin monomers
**4**	c338460_g1_i1_1	AA10	5.36	Annotated as a lytic polysaccharide monooxygenase
**5**	c63931_g1_i2_7	GH48	5.26	Annotated as a GH48 (cellobiohydrolase)
**7**	c64390_g1_i1_1	GH10/CBM2	4.33	Annotated as a GH10 (endo-/exo-xylanase) with CBM2 (cellulose/xylan binder)
**8**	c63105_g1_i1_1	GH5/CBM2	3.69	Annotated as GH5 (cellulase/xylanase) with CBM2 (cellulose/xylan binder)
**9**	c63931_g1_i1_7	GH48/CBM2	2.98	Annotated as a GH48 (cellobiohydrolase) with CBM2 (cellulose/xylan binder)
**11**	c143700_g1_i1_1	GH6	2.32	Annotated as GH6 (endoglucanase, cellobiohydrolase) but no CBM
**15**	c44042_g1_i1_2	PL1	1.38	Annotated as PL1 - Pectin lyase

**23**	c66145_g1_i1_1	CBM20	1.01	Annotated as containing three distinct CBM20 domains (Starch binding)
**31**	c64404_g1_i1_6	GH3	0.89	Annotated as GH3 (glucanase, xylosidase)
**33**	c62312_g2_i1_4	AA10	0.83	Annotated as a lytic polysaccharide monooxygenase
**40**	c65180_g3_i1_1	AA2	0.63	Annotated as containing three distinct AA2 domains (lignin peroxidase, manganese peroxidase)
**44**	c65093_g2_i2_8	GH9/CBM4	0.55	Annotated as GH0 (endo/exo-glucanase, cellobiohydrolase) with CBM4 (xylan and amorphous cellulose binder)
**57**	c139840_g1_i1_1	CE10	0.28	Annotated as CE10 – carbohydrate esterase family
**84**	c17840_g1_i1_8	CBM44	0.14	Annotated as 4x CBM44 domains: xylan/cellulose binding
**100**	c61378_g1_i1_7	CBM44	0.08	Annotated as 4x CBM44 domains: xylan/cellulose binding
**118**	c61637_g1_i1_4	CBM44	0.04	Annotated as 11x CBM44 domains, xylan/cellulose binding

(A)

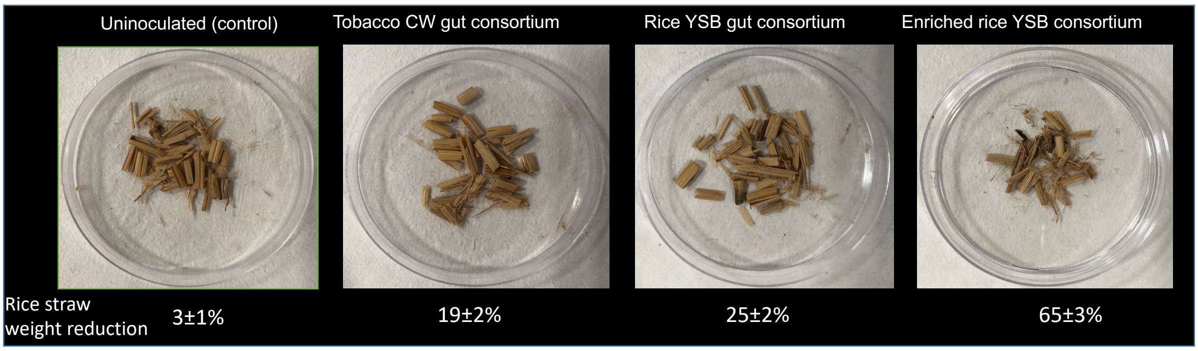

(B)

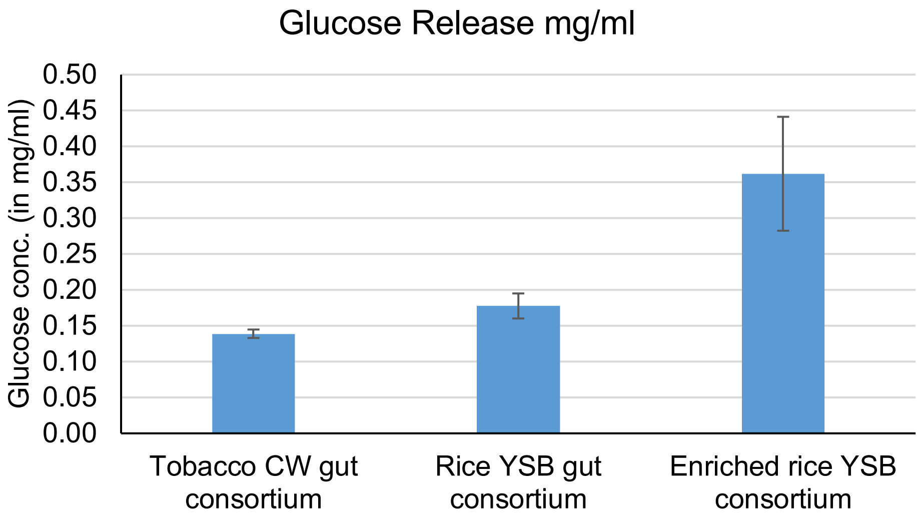

Figure S1 (a) Reduction in rice straw weight after 7 days of incubation with different gut consortium, with uninoculated medium as a control. (b) Glucose release after incubation of supernatant of different consortium with rice straw for 7 days.

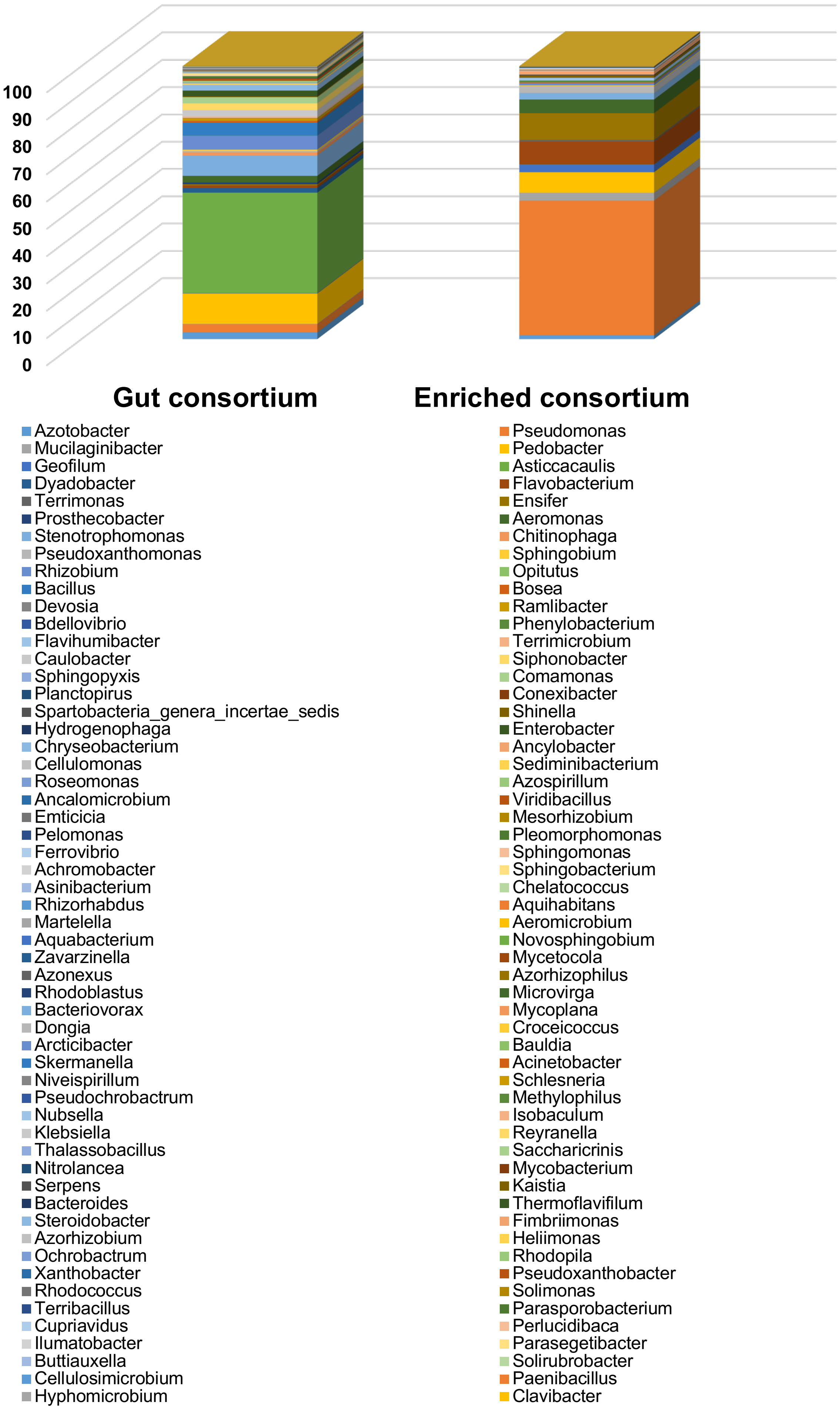

Figure S2 Change in community structure as a result of enrichment

a

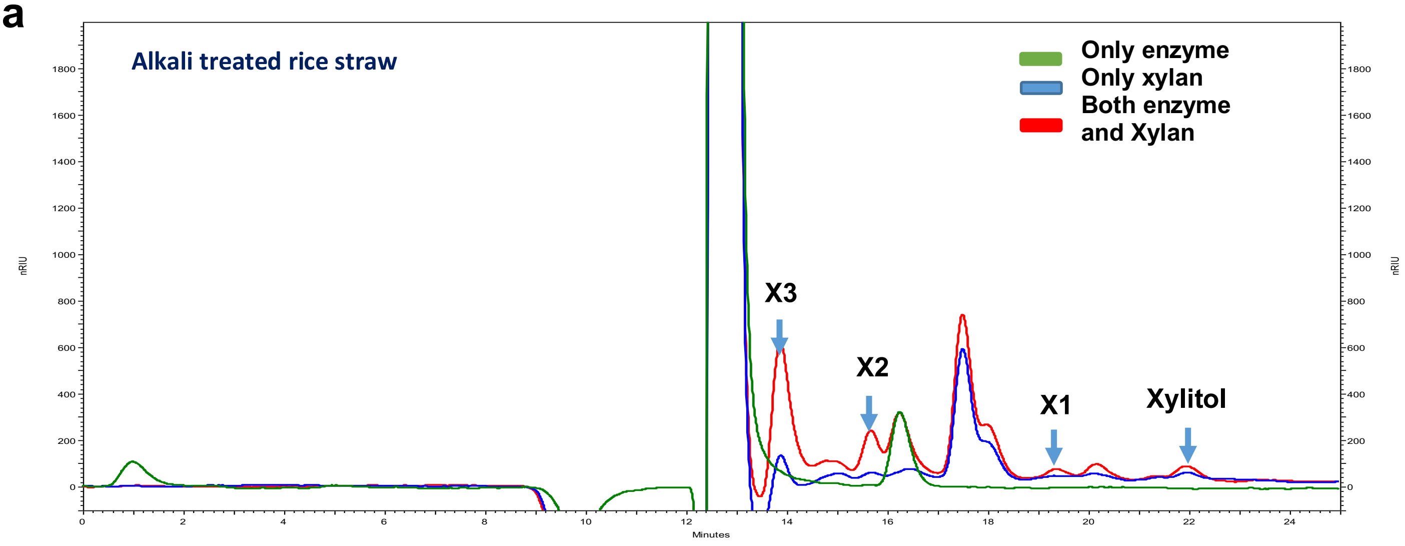

b

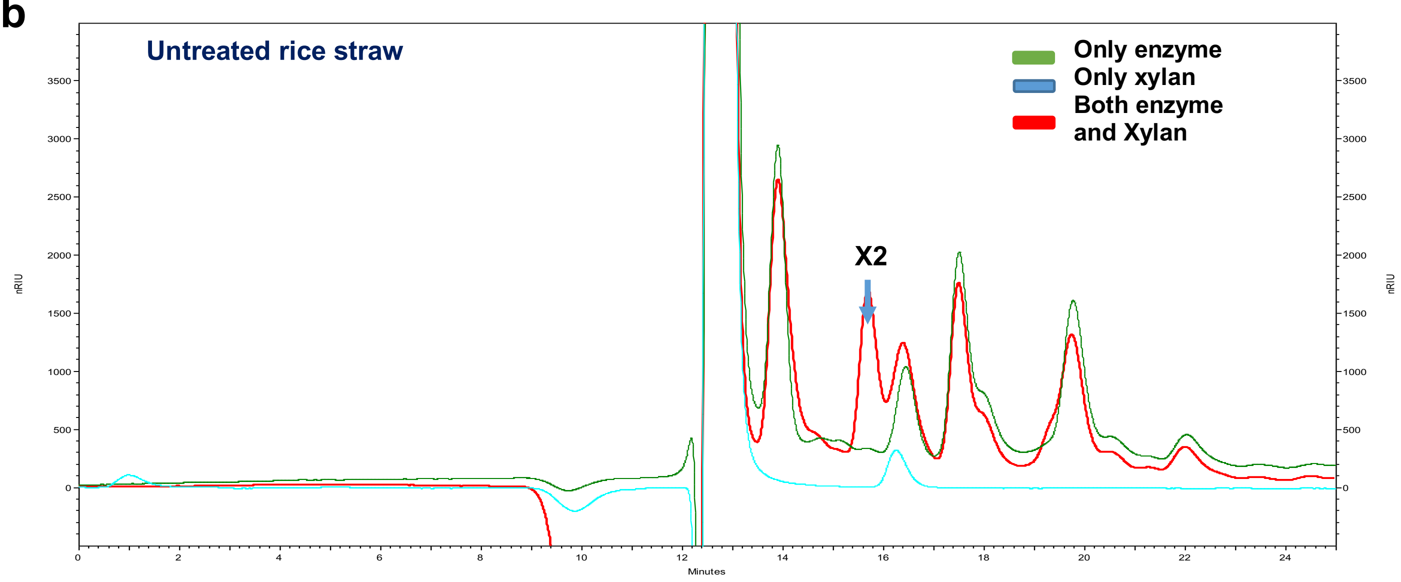

X1: Xylose; X2: Xylobiose; X3: Xylotriose

Figure S3 Alkali treated (a) and untreated (b) rice straw hydrolysis and product analysis
